# FIRST BRAZILIAN CONSENSUS ON MULTIMODAL TREATMENT OF COLORECTAL LIVER METASTASES. MODULE 1: PRE-TREATMENT EVALUATION

**DOI:** 10.1590/S0102-6720201500040002

**Published:** 2015

**Authors:** Felipe José Fernandez COIMBRA, Heber Salvador de Castro RIBEIRO, Márcio Carmona MARQUES, Paulo HERMAN, Rubens CHOJNIAK, Antonio Nocchi KALIL, Evanius Garcia WIERMANN, Sandro Roberto de Araújo CAVALLERO, Fabricio Ferreira COELHO, Paulo Henrique de Souza FERNANDES, Anderson Arantes SILVESTRINI, Maria Fernanda Arruda ALMEIDA, Antônio Luis Eiras de ARAÚJO, Marcos PITOMBO, Heberton Medeiros TEIXEIRA, Fábio Luiz WAECHTER, Fábio Gonçalves FERREIRA, Alessandro Landskron DINIZ, Giuseppe D'IPPOLITO, Maria Dirlei F. de Sousa BEGNAMI, Gabriel PROLLA, Silvio Márcio Pegoraro BALZAN, Thiago Bueno de OLIVEIRA, Luís Arnaldo SZULTAN, Javier LENDOIRE, Orlando Jorge Martins TORRES

**Affiliations:** From the following Societies: International Hepato-Pancreato-Biliary Association (BRZ CHPTR) - CB-IHPBA; Brazilian Society of Surgical Oncology - SBCO; Brazilian Society of Clinical Oncology - SBOC; Brazilian College of Digestive Surgery - CBCD; Brazilian College of Surgeons - CBC; American Hepato-Pancreato-Biliary Association - AHPBA, São Paulo, Brazil

**Keywords:** Colorectal cancer, Liver metastases, Brazilian consensus, Pre-treatment workout

## Abstract

**Background:**

: Liver metastases of colorectal cancer are frequent and potentially fatal event in the evolution of patients with these tumors.

**Aim:**

: In this module, was contextualized the clinical situations and parameterized epidemiological data and results of the various treatment modalities established.

**Method:**

Was realized deep discussion on detecting and staging metastatic colorectal cancer, as well as employment of imaging methods in the evaluation of response to instituted systemic therapy.

**Results:**

: The next step was based on the definition of which patients would have their metastases considered resectable and how to expand the amount of patients elegible for modalities with curative intent.

**Conclusion:**

: Were presented clinical, pathological and molecular prognostic factors, validated to be taken into account in clinical practice.

## Abstract (pt)

**Racional:**

: As metástases hepáticas de câncer colorretal são evento frequente e potencialmente fatal na evolução de pacientes com estas neoplasias.

**Objetivo:**

: Neste módulo procurou-se contextualizar esta situação clínica, bem como parametrizar dados epidemiológicos e de resultados das diversas modalidades de tratamento estabelecidas.

**Método:**

: Foi realizada discussão sobre como detectar e estadiar o câncer colorretal metastático, bem como o emprego dos métodos de imagem na avaliação de resposta ao tratamento sistêmico instituído.

**Resultado:**

: Fundamentou na definição de quais pacientes teriam suas metástases consideradas ressecáveis e de como se poderia ampliar a gama de pacientes submetidos às modalidades de tratamento ditas de intuito curativo.

**Conclusão:**

: Foram apresentados os fatores prognósticos clínicos, patológicos e moleculares com validação para serem levados em consideração na prática clínica.

## INTRODUCTION

Liver metastases of colorectal cancer are frequent and potentially fatal event in the evolution of patients with these malignancies. In this module was contextualize its clinical situation, as well as parameterize epidemiological data and results of the various established treatment modalities.

## METHOD

Discussion on detecting and staging metastatic colorectal cancer was performed, as well as the use of imaging methods in the evaluation of response to systemic treatment instituted.

## RESULTS

Topic 1 - Epidemiology and results of treatment in colorectal liver metastases (CLM)

Colorectal cancer (CRC) ranks fourth in global statistics of cancer incidence, with approximately 1,360,000 cases/year. With regard to mortality, it is estimated that there are over 693,000 deaths related to the disease in the world and it is the third leading cause of death in women and the fourth in men^1^. The number of new cases estimated for Brazil in 2014 was approximately 32,600 and it was the third most common cancer in men and the second among women, excluding non-melanoma skin cancers^2^. 

Approximately half of the patients with CRC develop metastases during their lives^3^^,^ 4 ^,^ 5 ^,^ 6 ^,^ 7 ^,^ 8. The most common site of metastatic CRC is the liver, occurring in 80% of cases^8^^,^ 9 ^,^ 10 ^,^ 11 ^,^ 12 ^,^ 13, representing approximately half of all patients with CRC^3^^,^ 4 ^,^ 5 ^,^ 6 ^,^ 7 ^,^ 8 and as the single site of metastasis in 20 to 50%^14^^,^ 15 ^,^ 16; however, only 15 to 30% are candidates for resection^12^^,^ 13 ^,^ 17. 

In population studies, the frequency of synchronous liver metastases from colorectal cancer CLM varies from 14.5 to 24%^8^^,^ 12 ^,^ 14 ^,^ 18 ^,^ 19 ^,^ 20 ^,^ 21. In a French population-based study with 24 years of follow-up of patients diagnosed with CRC, there was stability in the diagnosis of synchronous CLM during the period with crude incidence calculated at 11.3/100,000 for men and 6.9/100,000 for women, age-adjusted incidence at 7.6/100,000 and 3.7/100,000 respectively^8^. 

The frequency of metachronous CLM is highly variable in the literature, arising from database differences and diversity of definitions. In prospective and retrospective studies of referral centers, this rate reaches 35%^22^^,^ 23 ^,^ 24. In observational prospective studies and population studies, this frequency is lower, ranging from 5.7 to 16.3%^8^^,^ 14 ^,^ 18 ^,^ 19 ^,^ 23. A majority of CLM occurs in the first three years^8^^,^ 14 ^,^ 16 ^,^ 18 ^,^ 19. The incidence of CLM is approximately 4.3% at one year, 8.7% at two years, 12% at three years and 16.5% at five years after resection^8^^,^ 18.

An interesting point to note is that the incidence of CLM may be lower in patients with chronic liver disease such as steatosis^25^, virus B hepatitis and virus C hepatitis 26 ^,^ 27 ^,^ 28. In a meta-analysis of observational studies, there was a lower incidence of CLM (OR=0.26 [0.18 to 0.38]; p<0.0001) in patients with chronic liver disease^29^.

Attention must be paid to the fact that there are no specific Brazilian epidemiological studies to determine the proportion of patients with CRC who develop liver metastases. In addition, the Brazilian National Cancer Institute (INCA) estimates may be underestimated because of underreporting, besides the fact that data are collected only in some reference centers in Brazil, not representing the entire population.

Emphasizing the observation above, a tentative estimate made for the Brazilian population based on the incidence rates supplied by INCA for colorectal cancer in 2014, which is 32,600 new cases/year, one can suppose that around 16,300 (50%) patients have or will have CLM, of which 2,445 to 4,890 patients/year (15 to 30%) will be potential candidates for liver resections. 

Various modalities, either isolated or associated, can be used in the treatment of liver metastases. Liver resection showed benefit compared to unresectable patients, with 5-year overall survival of 55.2% versus 19.5% and a median overall survival of 65.3 months versus 26.7 months, respectively^30^. Unfortunately, recurrence rates after surgery can reach up to 70% of cases^31^^,^ 32.

Looking at the same resectable metastases, a study by the European Organization for Research and Treatment of Cancer (EORTC) evaluated the role of chemotherapy with perioperative FOLFOX4 regimen. This study showed an absolute increase in progression-free survival of 8.1% (33.2% vs. 42.4%, HR: 0.77, p=0.041) in eligible patients, with a greater number of complications for the group submitted to chemotherapy^32^. 

Other studies had the aim to show the benefit of adjuvant chemotherapy after resection. A meta-analysis encompassing three randomized clinical studies confirmed a gain in progression-free survival and disease-free survival, but benefit in overall survival was not reached^33^. 

However, in the setting of unresectable metastatic disease, chemotherapy has an unquestionable role. Studies have evaluated its role (without monoclonal antibody) and found a conversion rate for resectable tumors of approximately 13.5%^34^. Additionally, in tumors that became resectable, the 5-year survival was between 23% and 35%^34^^,^ 35 ^,^ 36, and 10-year survival around 27%^35^. When we add more drugs to the chemotherapy regimen, as in the FOLFOXIRI regimen, the conversion rate was increased to 36%, accompanied by median overall survival of 22.6 months^37^. 

In this same scenario of unresectable metastatic disease, cetuximab was evaluated when associated with the FOLFIRI or FOLFOX regimen. The resectability of liver lesions was achieved in 38% of patients. In addition, in a retrospective analysis of KRAS status, the resection rate increased to 60% in patients with wild-type KRAS treated with cetuximab^38^. In another study with only FOLFOX associated with or not with cetuximab, the overall and median 5-year survival was 30% and 24.4 months, respectively, with a complete resection rate of 25.7%. The median survival in patients undergoing complete resection was 46.4 months^39^. Studies with panitumumab showed similar results with median overall survival not yet reached in patients with complete resection^40^. More recent Phase II studies evaluating the role of targeted therapy, without restricting metastasis sites, showed median overall survival of 25 to 29.9 months^41^^,^ 42.

Another monoclonal antibody, not taking into account the RAS status, is bevacizumab, an antibody that binds to circulating VEGF-A increasing the efficacy of any cytotoxic active regimen^43^. First-line use showed an increase in overall and progression-free survival and response rate when combined with 5FU/leucovorin / irinotecan^44^^,^ 45 or only 5FU/leucovorin^45^ or capecitabine^46^^,^ 47. Combining oxaliplatin also showed an increase in progression-free survival^48^. The combination with FOLFOXIRI showed better progression-free survival and response rate, with one of the longest survival rates that has been reported so far in this scenario^49^. 

To understand the impact of liver metastases in patient survival, we can make a non-ideal comparison between the above studies presented and those that evaluated the role of the same treatments in non-metastatic disease, especially in patients with stage III tumors. Survival rates vary from 47% at three years when only surgery is offered^50^, 57% at five years when adjuvant chemotherapy with a 5-fluorouracil and leucovorin regimen is added^51^, and 72.9% at six years when oxaliplatin is associated with the previous regimen^52^.

In a non-ideal comparison, it is concluded that patients with CLM may have their chance of being alive at five years reduced by at least 50%. Therefore, liver metastasis are considered the leading cause of morbimortality in these patients^12^, accounting for at least two-thirds of disease-related deaths^3^. 

**Figure:**
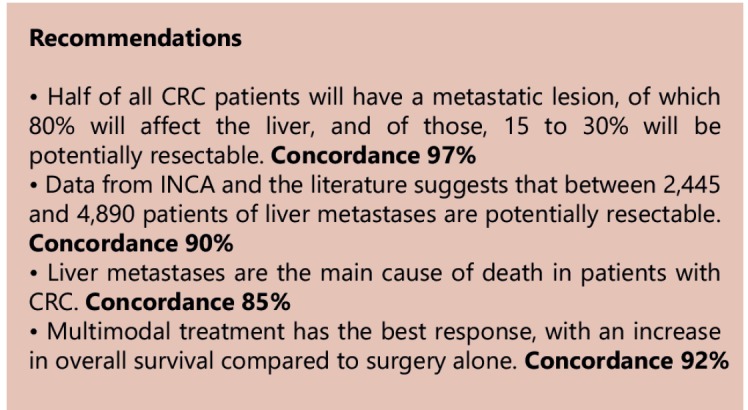

### Topic 2 - Diagnosis and staging of CLM

Imaging techniques that allow evaluation of liver metastases include ultrasound (US), computed tomography (CT), magnetic resonance (MRI) and positron emission tomography (FDG-PET)^53^^,^ 54. The modality of choice is determined by local availability and service experience.

### Transabdominal ultrasound

Despite being a method widely available and inexpensive, it exhibits low sensitivity rates and therefore has limited application in the evaluation of CLM. 

The overall sensitivity ranges between 50-77%, but it does not exceed 20% in lesions smaller than 1 cm.

Its main disadvantages: 1) operator-dependent method; 2) limited evaluation in obese patients with bowel distension or non-collaborative subjects.

The use of intravenous contrast (microspheres) increases the sensitivity for detection of focal liver lesions in about 20%, with results similar to those of CT with multidetectors^55^^,^ 56. However, this is a recently used technique with limited availability in Brazil.

### Computed tomography (CT) 

It is a widely available and relatively low-cost method; currently considered a standard technique for tumor staging, response evaluation and follow-up. 

The test should be performed in a multidetector-computed tomography (MDCT) with a dynamic study using intravenous iodinated contrast.

The limitations/disadvantages of the technique include exposure to ionizing radiation, risk of anaphylactic reactions to iodinated contrast and renal failure potential. 

The main diagnostic limitations are identification and characterization of focal hepatic lesions in livers with fat deposition^57^^,^ 58 and of sub-centimeter lesions^59^^,^ 60 ^,^ 61. 

### Magnetic resonance imaging (MRI) 

It is the most accurate imaging technique for the detection and characterization of focal liver lesions. However, costs are higher and it has restricted availability. Other limitations include magnetic field exposure and gadolinium use restrictions in patients with renal insufficiency.

The test may be performed using 1.5 or 3 Tesla equipment and the protocol should include sequences weighted in T1, T2, Diffusion (DWI) and volumetric T1 (3D) with dynamic study after contrast.

Dynamic study is usually performed with the administration of an extracellular distribution of gadolinium chelate, a hepatobiliary agent (disodium gadoxetato), that is available for use in Brazil. The hepatobiliary agent increases the detection rate of liver metastases^62^. 

Retrospective studies and recent meta-analyzes have demonstrated the superiority of MRI in the evaluation of liver metastases of colorectal carcinoma: 1) MRI showed superior sensitivity to TC both in analysis per patient (81.1 to 88.2% vs. 74.8 to 83.6%) and in analysis per lesion (80.3 to 86.3% vs. 74.4 to 82.6%); such superiority is related to higher detection of lesions smaller than 1 cm^57^^,^ 58; 2) MRI with conventional study + DWI + hepatobiliary contrast is the most sensitive and specific method for the characterization of LMCRC, especially in lesions smaller than 1 cm (sensitivity 94% and specificity 95%)^63^^,^ 64 ^,^ 65; 3) combined use of DWI and dynamic study with disodium gadoxetato significantly increases the diagnostic performance of MRI, with a detection rate higher than the isolated techniques^64^^,^ 65 ^,^ 66 ^,^ 67; 4) MRI with hepatobiliary contrast has greater accuracy than FDG-PET/CT in detection of small liver metastases (92% vs. 60%)^68^.

In a multicenter randomized prospective study, the performance of MRI with hepatobiliary contrast was superior to CT with iodinated contrast and MRI with extracellular gadolinium as first-line method in the initial evaluation of LMCRC^69^. 

### Positron emission tomography with fluorine-18 deoxyglucose (FDG-PET) 

It displays a very high sensitivity and specificity in the detection of liver metastases, with rates near 95%. Furthermore, it is useful to identify extra-hepatic metastases and local recurrence. However, its application is restricted due to low availability and high cost.

The main diagnostic limitations are in the detection of small pulmonary nodules and small liver metastases after chemotherapy 57 ^,^ 58 ^,^ 68.

Some studies have shown that in patients eligible for surgical resection of MHCR, FDG-PET/CT can identify extra-hepatic sites of metastases undetected by other methods, altering the therapeutic plan^70^^,^ 71 ^,^ 72. However, in a recent randomized clinical trial there was no significant change observed in surgical intent with the use of FDG-PE /CT compared to isolated MDCT^73^. 

### Intraoperative ultrasound (IOUS)

IOUS combined with surgical exploration is the gold standard method for detection of liver metastases and often alters the initial surgical plan^74^. 

It is an operator-dependent method and should be performed by a radiologist or surgeon experienced in the technique, using an intraoperative probe (5-12 MHz). In a study of 250 patients with preoperative evaluation performed with helical CT, IOUS detected additional hepatic lesions in 27% of patients^75^. Currently, even with the routine use of MDCT, benefits of IOUS are still observed, with changes to surgery in up to 20% of cases^76^^,^ 77. 

### Evaluation of systemic treatment response

The evaluation of response by imaging methods can be performed based on the following perspectives: 

###  Dimensional criteria 

The RECIST guideline criteria (version 1.1) is the most commonly used model for the evaluation of solid tumor response^78^.

###  Morphologic criteria

It was proven to be valid in cases of targeted therapy with bevacizumab. However, it was described in a study with high quality MDCT performed in a specialized center, and has yet to be validated in independent studies^79^. 

###  Functional methods

There is not enough evidence to support the routine use of FDG-PET and other functional techniques such as MRI with diffusion in CLM response evaluation^80^.

**Figure:**
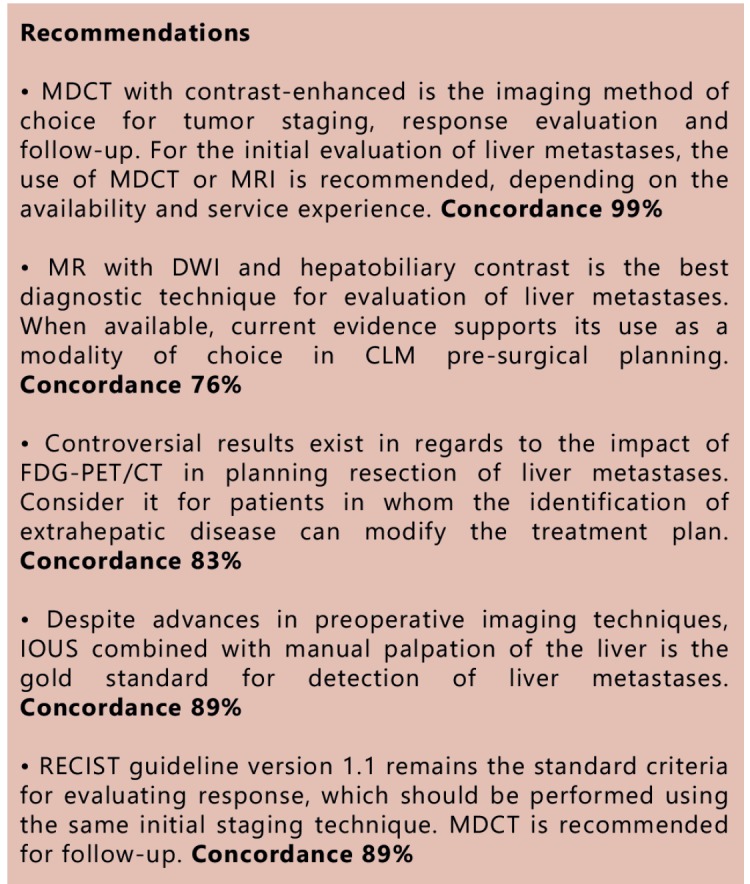

### Topic 3 - Definition of respectability

### How to estimate the mass/function of the future liver remnant 

###  Liver volumetry

The literature shows overlapping results in terms of residual liver mass estimate when compared to tomography and magnetic resonance. Ultrasonography has limitations inherented to the method, such as the interobserver variability. CT and MRI have shown similar results, but there are many more studies with CT, with further validated results^81^. Emphasis should be made to the correlation with volume measured in imaging and surgical weight of the resected liver, as it appears that both methods underestimate this result. The calculation of hepatic volume by CT and MRI is accurate and recommended for surgical planning, with similar results, using different correction factors^82^. CT - correction factor: 0.85; MRI - correction factor: 0.78. The main cause of discrepancy between liver volume calculated by CT and ex-vivo volume is blood perfusion and should be considered an overestimation in the order of 13%^83^. Hepatic volume by CT performed manually or automatically correlates strongly with actual liver volume. The automated way is faster^84^. Open and free software programs can be used by the surgeon to calculate the hepatic volume by CT with similar results to those obtained by the radiologist using dedicated software at workstations^85^.

Importantly, the estimates are only based on percentage of liver volume and are subject to limitation and should be viewed critically, especially in patients with hepatic steatosis/obesity and long courses of chemotherapy in the past. Some formulas have been developed and validated in search of greater security and should be used with caution especially in patients after portal vein embolization with modest growth^86^.

###  Anatomic, biological and clinical criteria of resectability

Resectability should be defined by an experienced surgeon in liver surgery^87^. The anatomical resection criteria include: complete resection of the tumor, absence of residual tumor, preserving at least one hepatic vein, homolateral maintenance to the portal pedicle and future live remnant >20%^88^. The recommended minimum margin at the time of resection is the macroscopic free margin. Positive microscopic margin can be accepted as an adverse finding in the postoperative period, but should not be offered as an option if imaging exams suggest that result^89^^,^ 90. R1 surgery offers survival similar to R0 resection in selected studies but it is still controversial^91^. 

A careful clinical evaluation should precede any liver surgery, particularly in patients with many comorbidities or the elderly. Note that resections in elderly patients over 70 years had similar results to those under 70 years old, with higher morbimortality in the first 90 days^92^. There are no studies that define the biological and clinical factors that represent criteria for resectability, but they are important prognostic factors and should be taken into consideration. They are: KRAS, NRAS, BRAF, CA 19-9, CEA, response to chemotherapy, number, size and location of metastases, synchronous or metachronous disease, presence of extrahepatic lesions, neutrophil-to-lymphocyte ratio, hypoechoic lesion on ultrasound, hTERT expression, disease-free interval, surgical margins, repeated resections^93^^,^ 94

### Strategies to increase respectability

#### Preoperative portal vein embolization (PPVE) 

Percutaneous PPVE increases the contralateral lobe with low complication rate and virtually no mortality for the procedure. The hepatectomy should be performed within three to four weeks after the embolization procedure^95^. Percutaneous PPVE should be indicated before hepatectomy when the surgical plan entails the removal of more than four liver segments and when future liver remnant (FLR) is: <20% in patients with normal liver; <30% in post-chemotherapy patients and <40% in cirrhotic patients^13^^,^ 96. Chemotherapy and anti-angiogenic inhibitors do not affect liver regeneration after portal vein embolization, but should be discontinued six weeks before the embolization procedure^97^. Even after PPVE, there is the occurrence of transient liver failure in about 2.5% of cases and acute liver failure in 1% of cases of major hepatectomies for colorectal cancer metastases. PPVE does not guarantee resectability, as 15% of patients fail to be operated on, in most cases due to the progression of neoplastic disease or inappropriate FLR growth^95^.

#### Two-stage hepatectomy

The indication of hepatectomy in two stages is uncommon and should be considered in initially unresectable patients with bilobar metastases, in whom resection at one time was not feasible because of insufficient FLR, even with the use of PPVE and ablative therapies. After the first stage of resection, 25% of patients will fail to reach the second stage due to disease progression in most cases. The second stage has twice the morbimortality of the first stage. Patients who complete the two stages may have similar survival to those who undergo just a single resection in their treatment^98^^,^ 99. Some recommendations on the surgical technique should be highlighted as: avoid leaving viable metastasis in FLR after the first stage, using radiofrequency ablation if necessary; avoid dissection of the pedicle in the first stage and mobilization of the lobe to be resected in the second stage^100^; resection of the primary tumor in the first stage in patients with synchronous metastases decreases the number of surgical procedures and facilitates chemotherapy^101^. Chemotherapy in the interval between the first and second stage does not guarantee lower rate of disease progression or a greater chance to complete the second stage^102^.

#### Associating liver partition and portal vein ligation for staged hepatectomy (ALPPS)

The ALPPS strategy must be performed by teams with experience in complex liver surgery^103^^,^ 104 ^,^ 105 ^,^ 106. During the stages of ALPPS, the association with major abdominal surgeries should be avoided^104^. The indication for ALPPS is resection with curative intent of large liver tumors with inadequate FLR volume and as an alternative to the classic strategy in two stages, especially as salvage surgery in patients undergoing portal embolization/ligation with insufficient gain of residual liver mass^107^^,^ 108 ^,^ 109. ALPPS is a technical option in patients with portal branch thrombosis that precludes percutaneous embolization^103^^,^ 110. The potential for tumor progression in the ALPPS strategy is at least the same as portal embolization^111^^,^ 112 ^,^ 113. However, ALPPS results in higher morbimortality rates as well as more severe postoperative complications in both surgical stages^103^^,^ 114 ^,^ 115. Hypertrophy of the residual liver provided by ALPPS (±75%) is similar to percutaneous portal embolization that extends to segment IV, and is significantly superior to isolated right portal embolization/ligation^115^. 

#### Radiofrequency associated with resection

Radiofrequency ablation (RFA) is no substitute for liver resection in the treatment of colorectal liver metastases, even in tumors smaller than 3 cm^116^. The indication of RFA associated with hepatic resection is rare, but its use occurs in 25% of patients who require repeated hepatectomy in the course of their treatment, and is associated with increased intrahepatic recurrence^117^. In patients with bilobar metastases where resection was indicated in combination with RFA, recurrence was similar in the ablation site, in wedge resection margin and segmental resection margin. Resection associated with RFA of more than 10 lesions is associated with shorter time to recurrence^118^. One should always seek an ablation area that provides a minimum margin of 1 cm beyond the tumor. Its ideal use is for tumors up to 3 cm in surgery, where resection is not viable and/or patients without performance status for surgery and when percutaneous portal vein is preferable.

**Figure:**
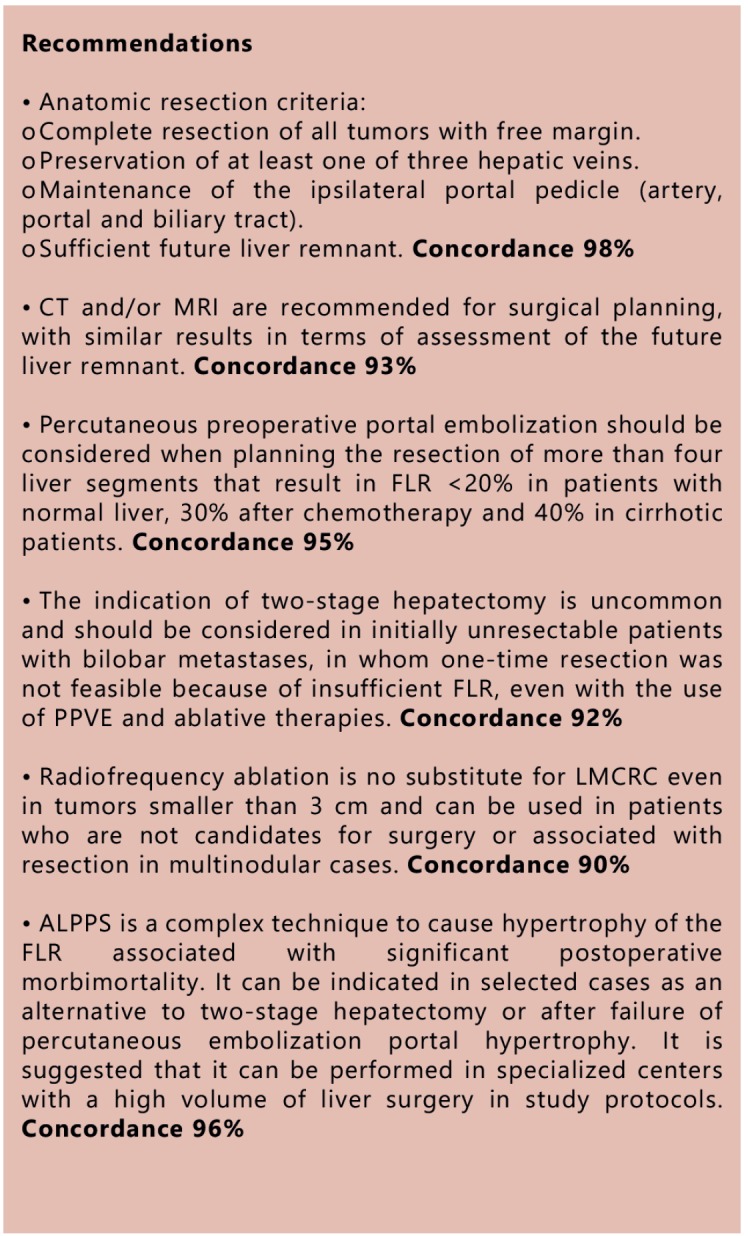

#### Topic 4 - Clinical, pathological and molecular prognostic factors in treatment definition 

There are clinical, pathological and molecular factors that can help estimate the prognosis of patients with LMCRC who undergo hepatectomy. These factors can be considered individually or in association with clinical risk scores. They are useful to understand the potential benefits and risks of recurrence, but should not be used to contraindicate surgical resection. Some prognostic factors such as margin, postoperative complications and pathological response to chemotherapy can only help estimate the benefit or risk after surgery.

#### Age and postoperative complications 

A study of 20,023 stage IV patients recruited in a randomized clinical trial (RCT) carried out by the ARCAD Clinical Trials Program database showed that younger and elderly patients had worse overall survival (OS) and progression-free survival (PFS)^119^. However, this study only analyzed patients treated with first-line palliative chemotherapy without analyzing the subgroup of patients undergoing resection of liver metastases.

In a retrospective study involving 806 patients undergoing hepatectomy in a single French center, 7% of patients had ≤40 years. Multivariate analysis showed that age ≤40 years was an independent prognostic factor associated with worse PFS^120^. 

In the Livermet Survey study with 7,764 patients, 20.9% were aged ≥70 years. Mortality at 60 days for patients ≥70 years was 3.8% vs. 1.6% for younger patients (p<0.001) and 3-year OS was 57.1% vs. 60.2% (p<0.001) respectively^121^. Therefore, resection of liver metastases in older patients has similar results to younger patients, with acceptable mortality.

A meta-analysis of four studies with 2,280 patients showed decreased 5-year DFS (OR 1.98) and OS (OR 1.68) for patients who had postoperative complications^122^.

#### Multiple liver metastases

The Memorial Sloan Kettering Cancer Center analyzed its database of patients who underwent resection of liver metastases between 1998 and 2002, and from a total of 584 patients, 98 (17%) had four or more liver metastases^123^. In this group of patients, median OS was 41 months and 5-year OS was 33%. However, median DFS was 14 months, 3-year and 5 year DFS were 12% and 0%, confirming the high risk of recurrence for patients with four or more liver metastases.

A retrospective Japanese study with 736 patients divided the patients into three groups: group A with 1-3 metastases ( *n* = 493 patients), group B with 4-7 metastases (n=141) and group C with eight or more metastases (n=102)^124^. OS at five years was 56% in group A, 41% in group B and 33% in group C. However, 5-year RFS was 29% for group A, 12% for group B and 1.7% for group C.

#### Meta-analysis of prognostic factors

A meta-analysis of survival after liver resection for metastatic colorectal cancer demonstrated a modest predictive relationship with survival; however, seven prognostic factors were significant: positive lymph node in the primary tumor, CEA level, extrahepatic disease, tumor grade, positive margins, more than one liver metastasis and tumor diameter greater than three centimeters^125^. Pooled effect calculated for these seven prognostic factors ranged from 1.52 to 2.02.

#### Early relapse in less than six months 

In a retrospective series of the Livermet Survey with 6,025 patients, 2,734 (45.4%) had recurrence, of which 639 (10.6%) had early recurrence^126^. OS at five years was 26.9% for patients with early recurrence vs. 49.4% (p<0.0001) for those who did not have it. Multivariate analysis demonstrated that the prognostic factors associated with early recurrence were: T3-4 tumors, synchronous metastases, more than three metastases, positive microscopic margin (R1 resection) and the use of radiofrequency ablation (RFA). 

####  Clinical risk scores

Clinical risk scores and nomograms are intended to estimate the benefit of liver resection correlated with prognostic factors of survival 127 ^,^ 128 ^,^ 129 ^,^ 130 ^,^ 131 ^,^ 132 ^,^ 133. For example, Fong´s liver score criteria are node-positive primary tumors, DFS less than 12 months, more than one node, metastasis larger than five centimeters and CEA above 200 ng/mL^127^. The presence or absence of each of these factors leads to a score from 0 to 5, which correlated with with 5-year OS. Most clinical risk scores are rarely used and the lack of external validation of these risk calculations prevent their use in selecting patients eligible for liver resection. 

#### Pathological response to preoperative chemotherapy 

Retrospective studies demonstrate that pathological response to preoperative chemotherapy, with variable definitions of response from one study to another, correlate with improved OS^134^^,^ 135.

#### Resection margins

Several retrospective studies demonstrate that positive margins are associated with increased risk of recurrence in the surgical margin, but that complete resection and not the millimeter size of the margin is what is more important^89^^,^ 91 ^,^ 136 ^,^ 137. A meta-analysis of 18 studies with 4,821 patients showed that negative margins ≥1 cm are superior to negative margins <1 cm in 5-year OS (46% vs. 38%, p=0.009)^138^.

In a prospective observational study of 2,715 patients, positive margin was defined as the distance between metastasis and the border of resection less than one millimeter and negative margin as margin more than 1 mm. In this study, DFS at three years in patients with margin greater than 1 mm was significantly superior to that of cases with a shorter margin and there was no additional gain in DFS with margins greater than 1 mm^139^.

#### KRAS, NRAS and BRAF

KRAS and NRAS are predictors of therapy results with anti-EGFR, but they have a less established role as a prognostic factor in metastatic colorectal cancer^140,141,142^. A retrospective analysis of a study with 202 patients suggests KRAS as a possible prognostic factor after surgery for liver metastases (HR 1.99)^134^. However, BRAF is a strong adverse prognostic factor in metastatic colorectal cancer and also post-metastectomy^143,144^. 

There is a strong agreement (>90%) in RAS/BRAF results between primary tumor and metastasis and therefore the test can be done in both biopsies of the primary tumor and in metastases biopsies^145,146^.

It is recommended that the report should contain: 1) type of test performed and sensitivity; 2) type of material tested (primary tumor or metastases); 3) type of extraction (macro or laser) and the percentage of tumor represented; 4 ) mutated codon and the type of mutation; 5) cut-off used in the laboratory for the interpretation of the results. 

**Figure:**
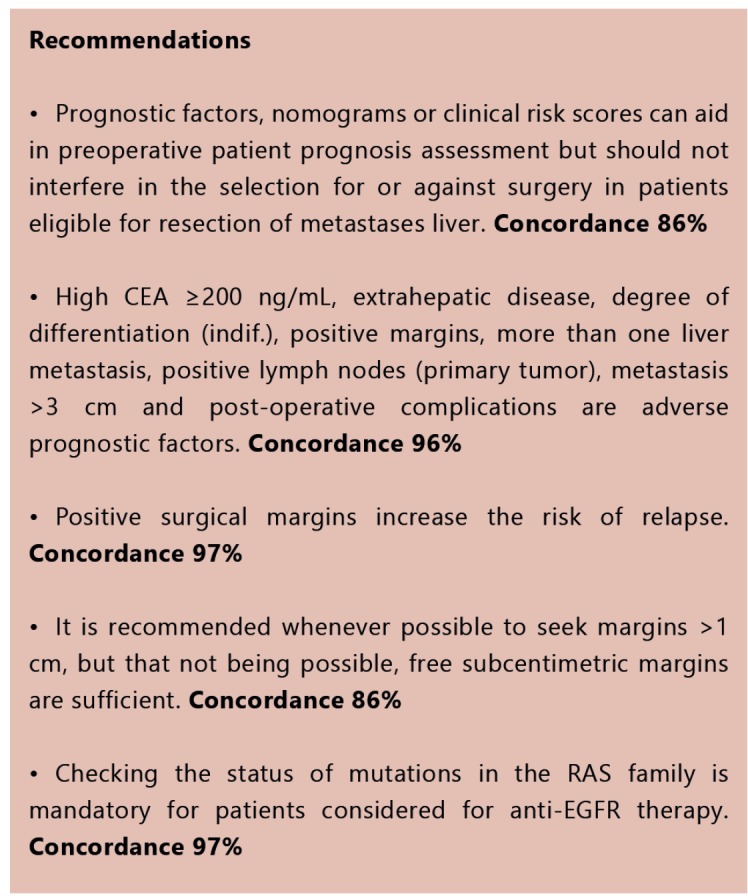

## CONCLUSION

Clinical, pathological and molecular prognostic factors with validation were presented to be taken into account in clinical practice.
